# Fermentation of ginkgo biloba kernel juice using *Lactobacillus plantarum* Y2 from the ginkgo peel: Fermentation characteristics and evolution of phenolic profiles, antioxidant activities *in vitro*, and volatile flavor compounds

**DOI:** 10.3389/fnut.2022.1025080

**Published:** 2022-10-28

**Authors:** Jie Yang, Yue Sun, Jinling Chen, Yu Cheng, Haoran Zhang, Tengqi Gao, Feng Xu, Saikun Pan, Yang Tao, Jing Lu

**Affiliations:** ^1^Co-Innovation Center of Jiangsu Marine Bio-industry Technology, Jiangsu Ocean University, Lianyungang, China; ^2^Jiangsu Key Laboratory of Marine Bioresources and Environment/Jiangsu Key Laboratory of Marine Biotechnology, Jiangsu Ocean University, Lianyungang, China; ^3^College of Food Science and Technology, Nanjing Agricultural University, Nanjing, China

**Keywords:** ginkgo biloba kernel juice, lactic acid bacteria fermentation, phenolic substances, free amino acids (FAAs), volatile flavor substances

## Abstract

In this study, a strain of *Lactobacillus plantarum* Y2 was isolated from the ginkgo peel, and showed adequate adaptation to the ginkgo biloba kernel juice. After 48 h of fermentation, the number of viable cells in the stable growth phase was remained at 10.0 Log CFU/mL, while the content of total organic acid increased by 5.86%. Phenolic substances were significantly enriched, and the content of total phenolic substances increased by 9.72%, and the content of total flavonoids after fermentation exceeded 55.33 mg/L, which was 3.6 times that of the unfermented ginkgo juice. The total relative content of volatile flavor compounds increased by 125.48%, and 24 new volatile flavor substances were produced. The content of total sugar, total protein, and total free amino acid decreased to 44.85, 67.51, and 6.88%, respectively. Meanwhile, more than 82.25% of 4′-*O*-methylpyridoxine was degraded by lactic acid fermentation, and the final concentration in ginkgo biloba kernel juice was lower than 41.53 mg/L. In addition, the antioxidant and antibacterial activities of fermented ginkgo biloba kernel juice were significantly enhanced. These results showed that LAB fermentation could effectively improve the nutritional value and safety of ginkgo biloba kernel juice.

## Introduction

*Ginkgo biloba* L., one of the oldest living tree species on earth (1), has been reported to have great development potential in the field of functional food (2). Meanwhile, ginkgo biloba kernel juice contains various active ingredients such as ginkgo phenolic acids, flavonoids, and polysaccharides, etc., making it widely used in food, health products, cosmetics, medicine and other fields (3). In particular, the special physiologically active components such as flavonoids and ginkgoid acid contained in it have the functions of anti-oxidation, anti-aging, anti-inflammatory, anti-allergic, and inhibiting nerve cell apoptosis (4). Recent studies on ginkgo have mainly focused on ginkgo leaves and relatively few studies on ginkgo kernels (5). Therefore, it is necessary and meaningful to further develop and utilize ginkgo kernels.

4′-O-methylpyridoxine (MPN) is a vitamin 6 derivative which can cause in pregnancy, radiation sickness, seborrheic dermatitis, and other pathologies. Interestingly, only a threshold concentration of MPN is beneficial to the human body; a higher concentration can lead to toxic reactions in the human body. The MPN content in medicine ranged from 0 to 9.77 μg/mL (6), and in ginkgo kernels ranged from 172.8 to 404.2 μg/g, indicating that excessive consumption of ginkgo kernels was harmful to human health (7). As MPN concentrations severely limit the development and utilization of ginkgo kernels, MPN degradation during the production process of ginkgo is a vital concern that needs to be addressed.

Lactic acid bacteria (LAB) have been widely used in the fermentation of pickles, yogurt, soy sauce and tempeh, owing to their ability to regulate the human intestinal flora, promote the absorption of nutrition substances, kill harmful flora and the toxins, and ameliorate food flavor (8). Moreover, the secondary metabolites in the fermentation process with LAB have several health benefits, such as promoting the activity of antioxidant enzymes in cells, and increasing the content of beneficial substances. For example, Verón found that LAB fermentation could increase the activities of ferulic acid, caffeic acid derivatives and intracellular antioxidant enzymes, and enhance the overall antioxidant capacity of the fermentation broth (9).

Currently, studies on ginkgo mainly focuses on ginkgo leaves, while there are relatively few studies on ginkgo kernels. Ginkgo products mainly include canned ginkgo kernel, ginkgo wine, and beverages (10, 11). However, only a few studies had been conducted on fermented silver almonds. In this study, a LAB strain was selected from ginkgo peel to ferment ginkgo biloba kernel juice. The physiological and biochemical indices of fermented ginkgo biloba kernel juice were analyzed, and the antioxidation activity and bacteriostatic ability were evaluated. The results of this study will enrich the physiological and biochemical studies of LAB-fermented ginkgo biloba kernel, and improve the reference for the development of ginkgo products.

## Materials and methods

### Screening and identification of lactic acid bacteria strains from the surface of ginkgo peel

Ginkgo was sourced from the campus of Jiangsu Ocean University (Lianyun Gang, Jiangsu province), and the LAB strains were screened from the surface of the ginkgo fruit. The ginkgo fruit was inoculated in MRS culture medium (g: mL, 1:1.5) and cultured at 37°C for 24 h. The fermentation broth was spreaded on MRS plates to obtain single colonies which were underlined on MRS plates and placed in a 37^°^C incubator for 24 h to obtain single colonies. The obtained single colonies were then grown in MRS medium, with 40% glycerol stocks stored and frozen in a −40^°^C refrigerator. These LAB strains were identified using 16S-rRNA, physiological and biochemical tests in accordance with conventional methods (12).

### Preparation and fermentation of ginkgo biloba kernel juice

The ginkgo fruit was boiled for 5–10 min, and the shell and seed coat were removed. After washing with water, the shelled ginkgo kernels were crushed and broken up in a certain proportion (ginkgo biloba kernels: distilled water = 1:2.2, g:mL), and the starch dissolution rate was required to reach more than 80%. The α-amylase (20 U/g, ginkgo mass) and saccharification enzyme (30 U/g, ginkgo mass) were added, and then ginkgo biloba kernel juice was saccharified at 50^°^C for 2 h. After the enzymatic hydrolysis the ginkgo biloba kernel juice was completed. It was filtered through a 100-mesh filter, and pasteurized in a water bath at 90°C for 20 min (13). In a 500 mL sealed Erlenmeyer flask, 1% (v/v) inoculum of ginkgo biloba kernel juice was inoculated to ensure an initial viable count of approximately 5.0 Log CFU/mL. The fermentation process was performed in an incubator at 37°C for 48 h. At the end of fermentation, the bacterial cells were removed by centrifugation (10,000 *g*, 10 min, 4°C), and the supernatant was collected for further chemical analysis.

### Determination of viable cell count, pH and antibacterial activity of fermented ginkgo biloba kernel juice

Viable cell counts were determined using the standard plate method (14). The fermentation broth was first diluted to an appropriate concentration, then the diluted liquid was spread on a plate and the colonies that grew were counted. A precision pH meter (PHS-3C, Shanghai INESA Scientific Instrument Co., Ltd., Shanghai, China) was used to measure the pH value of each sample of ginkgo biloba kernel juice fermentation broth and the bacteriostatic activity was assessed by measuring the diameter of the outward inhibition zone of the Oxford cup using a Vernier caliper (15). The centrifuged supernatant (10 mL) was concentrated in vacuo to 2 ml, and then screening through a 0.22 μm membrane screening. The bacterial pathogens were *Escherichia coli* CICC 10003, *Staphylococcus aureus* CICC 23656, and *Bacillus cereus s* CICC 23828.

### Determination of total sugar content and total protein content of fermented ginkgo biloba kernel juice

The total sugar content was determined using the sulfuric acid phenol method (16). The principle of this method was that polysaccharides were hydrolyzed into monosaccharides under the action of sulfuric acid, and then rapidly dehydrated to form uronic derivatives, which were then combined with phenol to form orange-yellow compounds. The absorbance values at 470 nm of the orange-yellow compounds were linearly related to the monosaccharide concentration. A standard curve was established using anhydrous glucose and the results were expressed in μg/mL of glucose equivalents. Protein was detected using the Coomassie brilliant blue method G250 (17). A standard curve was established using a Bovine Serum Albumin (BSA) standard solution, and the total protein equivalent was expressed in mg/mL.

### Determination of organic acid and free amino acid content of fermented ginkgo biloba kernel juice

The organic acid spectrum was analyzed using a the Shimadzu LC-2010A system (Shimadzu, Tokyo, Japan), following the method of Luo Ke with some modifications (18). The chromatographic column was an Agilent TC-C18 column (4.6 × 25 mm, 5 μm), the detection wavelength was 210 nm, 0.08 M KH_2_PO_4_ solution (adjusted to pH 2.5 with phosphoric acid) was used for isocratic elution. The column temperature was 30°C, the flow rate was 0.7 mL/min, and the injection volume was 20 μL. The free amino acids were quantified using Adeyeye’s method with slight modifications. The analytical column was 2622PH 4.6 mm I.D. × 60 mm, the flow rate was 0.40 mL/min, the column temperature was 57°C, the reaction temperature was 135°C, and the detection wavelength was 570 nm, and the injection volume was 20 μL.

### Determination of phenolic content of fermented ginkgo biloba kernel juice

The total flavonoids was determined according to the method of Kwaw et al. (19). A calibration curve was constructed with rutin and the results were expressed in μg/mL of rutin equivalents. The total phenolic content of crude polyphenols in fermented or unfermented ginkgo biloba kernel was determined using the Folin-Ciocalteu method. A calibration curve constructed using gallic acid and the results were expressed in mg/L of gallic acid equivalents. Phenolic acid and flavonoids content were determined according to the method described by Chiang et al. (20). The column was Inertsil ODS-3 5 μm (4.6 × 250 mm, 5 μm), and the column temperature was 25^°^C. The mobile phase was composed of solution A (1% acetic acid in water) and the solution B (1% acetic acid in methanol), and the flow rate was 0.6 mL/min. The gradient set was as follows: 0–10 min, 10–26% B; 10–25 min, 26–40% B; 25–45 min, 40–65% B; 45–55 min, 65–95% B; 55–58 min, 95–10% B; 58–65 min, 10% B. The wavelength for determination of phenolic acids and flavonoids was 280 and 350 nm, respectively. The concentration of each phenolic compound was calculated according to the standard calibration curve, and the results were expressed in mg/mL.

### Evaluation of antioxidant activity of fermented ginkgo biloba kernel juice

Total antioxidant capacity was measured in the same way as previously described (21). The total antioxidant capacity was calculated as follows: total antioxidant capacity = (Measured OD − Control OD)/0.01/30 × total reaction volume/sampling volume × sample dilution ratio before testing. The ABTS^+^-SA assay was performed using the method described by Tao et al. (21). Trolox was used as the standard, and the results were expressed in mmol/LTrolox equivalents. The Iron Reducing Antioxidant Ability (FRAP) of the fermented samples was evaluated by the method of Yan et al. (22).

### Determination of volatile flavor compounds and 4′-*O*-methylpyridoxine content of fermented ginkgo biloba kernel juice

The volatile components were separated, collected, and analyzed using a triple quadrupole GC-MS (Trace 1310/TSQ 9000, Thermo Scientific) (23). The headspace vial was filled with nitrogen to expel the air in the vial, and 5 mL tested sample was taken into the Thermo RSH autosampler for extraction. The extraction head was placed in the gas chromatography injection port, the injection port temperature was 250°C, the aging was 30 min, the oven temperature was set to 80°C, the water bath was set for 20 min, the adsorption extraction was 30 min, and the extraction head was inserted into the injection port for analysis for 5 min. The MPN content was determined reference to the method described by Yoshimura using the Agilent 1260 fluorescence detector (detection wavelength was 291 nm) (24).

### Statistical analysis

All experiments were performed three times in the same way, and all samples were analyzed in triplicate. The significance analysis was performed using SPSS Statistics 20 (IBM Corp., NY, USA), and the significance was assessed at *p* < 0.05 value. The principal component analysis (PCA) was performed using Origin 2018 (Origin Lab Corp., UK), and all data results were expressed as mean ± standard deviation.

## Results and discussion

### Evaluation of acid-producing capacity and antibacterial activity of ginkgo biloba kernel juice fermented by lactic acid bacteria strains from the surface of ginkgo fruit

**FIGURE 1:**
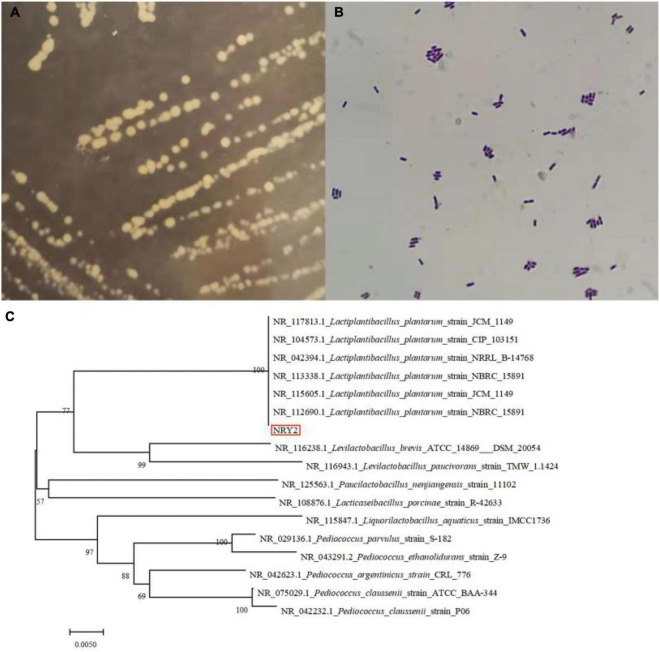
The colonial morphology (**A**, MRS plate), gram staining picture **(B)** and colonial morphology the hylogenetic tree **(C)** of *Lactobacillus plantarum* Y2.

In this study, nine LAB strains were isolated from the surface of ginkgo fruit. The developmental tree established by the BLAST program and the results of the physiology and biochemistry of the strains confirmed that all the selected strains were all *Lactobacillus plantarum* (Table 1 and Figure 1). Next, the acid-producing and antibacterial activities of the nine strains were evaluated. The pH of ginkgo biloba kernel juice, fermented by different strains, decreased, indicating that these strains had good acid production capacity. Among them, the pH of ginkgo biloba kernel juice fermented by the strain Y2 had a lower pH than the other strains, indicating that Y2 has a stronger growth and metabolism in ginkgo biloba kernel juice (25). In addition, the pH of ginkgo biloba kernel juice fermented by the strain Y2 had the best antibacterial effect on pathogenic bacteria (*E. coli*, *S. aureus*, and *B. cereus*), with inhibition zone diameters of 13.77 ± 0.32, 21.54 ± 0.58, and 15.57 ± 0.53 mm, respectively. When the acidity of fermented ginkgo biloba kernel juice is high, the pH value of the medium decreases, reducing conductivity for pathogenic bacterial growth (26). Lactic acid and compounds such as bacteriocins and synthetic hydrogen peroxide produced by the fermentation of lactic acid bacteria inhibit microbial growth (27). In conclusion, the strain Y2 had a good growth and metabolic status in ginkgo biloba kernel juice, and was selected as an effective fermentation strain for further experiments.

**TABLE 1:** The pH change and antibacterial activity determination of Ginkgo biloba kernel juice fermented by LAB strains used in this study.

Strain	Species	pH	Diameter of the inhibition zone (mm)		
			*E. coli*	*S. aureus*	*B. cereus*
Y1	*L. plantarum*	4.13 ± 0.03^a^	13.10 ± 0.10^ABab^	10.65 ± 0.35^Bb^	13.05 ± 0.05^Ab^
Y2	*L. plantarum*	4.03 ± 0.02^b^	13.20 ± 0.10^Ba^	13.05 ± 0.05^Ba^	15.15 ± 0.15^Aa^
Y3	*L. plantarum*	4.13 ± 0.03^a^	10.85 ± 0.15^Ab^	10.65 ± 0.35^Aab^	11.95 ± 0.05^Ab^
Y4	*L. plantarum*	4.15 ± 0.04^a^	12.30 ± 0.20^Ab^	12.05 ± 0.05^Aab^	13.85 ± 0.15^Ab^
Y5	*L. plantarum*	4.07 ± 0.07^a^	9.75 ± 0.25^Ab^	9.70 ± 0.30^Aab^	11.30 ± 0.30^Ab^
Y6	*L. plantarum*	4.08 ± 0.06^a^	11.80 ± 0.20^Ab^	10.40 ± 0.40^Aab^	12.70 ± 0.20^Ab^
Y7	*L. plantarum*	4.12 ± 0.01^a^	11.80 ± 0.20^Ab^	10.90 ± 0.10^Aab^	13.05 ± 0.05^Ab^
Y8	*L. plantarum*	4.09 ± 0.01^a^	12.45 ± 0.15^Ab^	12.10 ± 0.10^Aab^	11.80 ± 0.20^Ab^
Y9	*L. plantarum*	4.10 ± 0.01^a^	9.60 ± 0.20^Bab^	12.90 ± 0.10^Cb^	13.05 ± 0.05^Ab^

The diameter of the inhibition zone is greater than 15 mm as strong; 10–14 mm as medium; less than 10 mm as weak. Capital letters indicate significant differences between samples in the same row (*p* < 0.05). Lowercase letters indicate significant differences between samples in the same column (*p* < 0.05).

### Changes of viable cell count, pH value and antioxidant ability during strain Y2 fermentation

**FIGURE 2:**
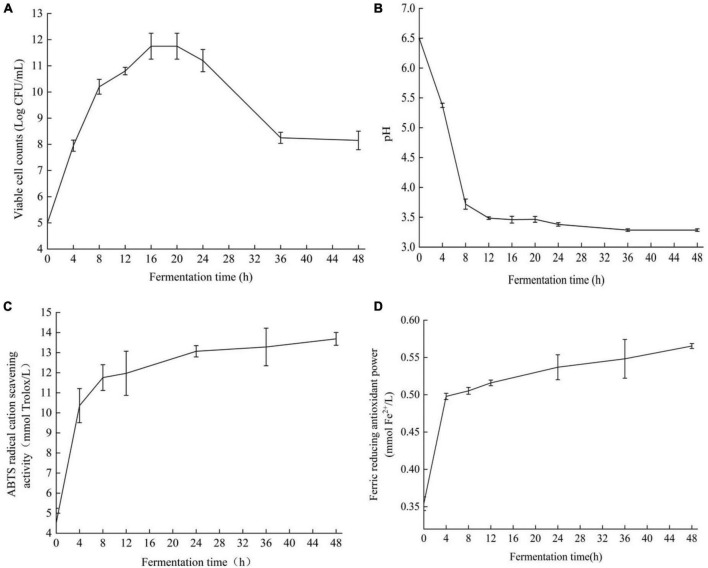
Viable cell count **(A)**, changes of pH **(B)**, ABTS^+^ free radical scavenging ability **(C)**, FRAP reducing antioxidant capacity **(D)** of Ginkgo biloba kernel juice fermented by strain Y2.

Cell viability is a functional feature used to evaluate bacterial growth (28). The strain Y2 grew well in ginkgo biloba kernel juice without any nutritional supplements according to the number of viable cells of strain Y2 in the fermenting process, showing that ginkgo biloba kernel juice could be used as a fermentation substrate (Figure 2A). The strain Y2 grew rapidly in the logarithmic phase from 4 to 8 h, and the number of viable bacteria increased significantly, indicating that Y2 was suitable for growth in ginkgo biloba kernel juice. The number of viable cells started at 5.0 ± 0.02 log CFU/mL and grew rapidly to 10.0 ± 0.03 log CFU/mL after 8 h of fermentation. After 24 h of fermentation, the number of viable cells stabilized at 12.1 ± 0.08 log CFU/mL. After 48 h of fermentation, the number of viable cells of LAB Y2 remained at 8.4 ± 0.03 log CFU/mL. The initial pH of ginkgo biloba kernel juice was 6.2 ± 0.04 (Figure 2B). In the early stage of fermentation, a large amount of lactic acid was produced, and the pH value of the fermentation broth decreased rapidly. After 12 h of fermentation, the rate of pH continued to gradually slow down reaching a final pH of 3.27 ± 0.07 at 48 h. According to the characteristics of lactic acid bacteria fermentation and acid production, Y2 was a LAB with strong acid production ability and good fermentation effect. The changes in antioxidant activity during the fermentation of ginkgo biloba kernel juice were shown in Figures 2C,D. The ABTS^+^ radical scavenging capacity and FRAP reducing capacity increased with time. After 48 h of fermentation, the free radical scavenging rate of ABTS^+^ increased from 4.52 ± 0.72 to 13.69 ± 0.32 mM, and the reducing capacity of FRAP increased from 0.35 ± 0.01 to 0.62 ± 0.02 mM. After 0–4 h of fermentation, the ABTS^+^ free radical scavenging rate and FRAP reducing ability increased significantly, and the antioxidant activity of the fermentation broth improved significantly. After 24 h of fermentation, the lifting speed approached slowly, and the antioxidant activity was basically stable at the end of fermentation.

### The consumption of total sugar content by Y2 fermentation and its influence on the bioactive components of organic acids

Carbon source catabolism could provide energy for the growth of *Lactobacillus* and indirectly promote an increase in organic acid content (29). The total sugar content decreased significantly from 12 to 36 h of fermentation, indicating that the lactic acid bacteria in the stable growth period consumed large amounts of carbon sources (Table 2). As fermentation entered the decay period (36–48 h of fermentation), lower substrate pH and reduced nutrients limited the growth of Y2 and indirectly affected its sugar metabolism, resulting in a stabilization of the total sugar contents (30). The consumption rate exceeded 44.85% after the fermentation ended.

**TABLE 2:** Total sugar, total protein and total phenolic content of Ginkgo biloba kernel juice fermented by strain Y2.

Fermentation time (h)	Total sugar content (μ g/mL)	Total protein content (mg/mL)	Total phenolic content (mg/L)
0	190.78 ± 2.04^a^	12.77 ± 1.14^a^	116.51 ± 0.51^b^
4	187.40 ± 0.63^a^	10.72 ± 1.04^b^	92.51 ± 2.51^d^
8	185.90 ± 0.13^a^	8.02 ± 1.45^c^	104.17 ± 3.33^c^
12	185.03 ± 0.25^b^	7.61 ± 0.04^c^	104.67 ± 0.58^c^
24	132.90 ± 4.08^c^	7.34 ± 0.13^c^	105.01 ± 1.01^c^
36	108.90 ± 0.25^d^	7.29 ± 0.27^c^	126.17 ± 0.77^a^
48	105.25 ± 3.24^d^	4.15 ± 0.19^d^	127.83 ± 0.28^a^

Results are expressed as the mean ± standard deviation of three replicates. The mass concentrations of substances in different time periods are represented by different letters, indicating significant differences (*p* < 0.05).

**FIGURE 3:**
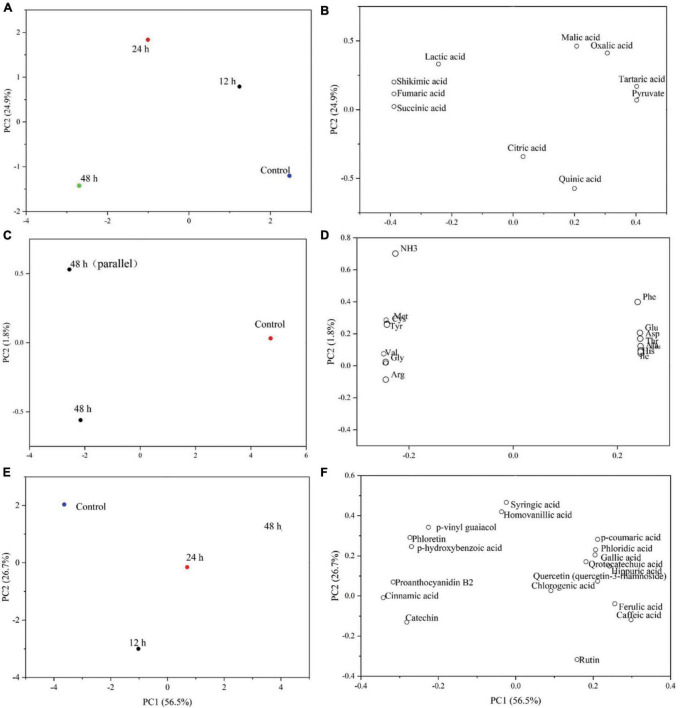
Principal component analysis of changes in organic acids **(A,B)**, free amino acids **(C,D)** and phenolic acids **(E,F)** during fermentation of Ginkgo biloba kernel juice by Y2 for 48 h.

During the fermentation, the organic acids were transformed into each other over time, and the total organic acid content increased by about approximately 5.86% after 48 h of fermentation. The components of all fermentation time periods were well separated from the control samples according to the component map (Figure 3A), showing that the fermentation of Y2 significantly changed the organic acid composition in ginkgo biloba kernel juice. According to the loading diagram (Figure 3B), malic acid, tartaric acid, citric acid, pyruvic acid and quinic acid all had high positive values for PCI, indicating that the content of these substances decreased during the fermentation process. While the decrease in malic acid was particularly obvious, levels of oxalic acid did not observe any changes during fermentation. Lactic acid, shikimic acid, fumaric acid, and succinic acid had high negative values for PCI, reflecting a general increase in content after fermentation.

Ginkgo kernel was rich in a variety of natural organic acids, of which malic acid had the highest content (926.91 ± 5.35 μg/mL), followed by pyruvic acid (80.27 ± 0.27 μg/mL), citric acid (43.77 ± 0.55 μg/mL) and tartaric acid (632.34 ± 19.98 μg/mL) (Table 3). Lactic acid was the main organic acid produced by the consumption of sugars by lactic acid bacteria in ginkgo biloba kernel juice, and the lactic acid content continued to increase throughout the fermentation process. The initial lactic acid content was 403.33 ± 17.77 μg/mL and increased to 722.58 ± 29.76 μg/mL after 48 h of fermentation. The production of abundant lactic acid reduced the pH value of the fermentation substrate. Pyruvate and citric acid could be decompose into various products such as lactic acid and acetic acid during fermentation (31). Malic acid, which was a good carbon source, observed the highest proportion of total organic acids. The content of malic acid decreased by about 25.25% during the fermentation process, while the content of lactic acid and succinic acid increased. Tartaric acid could be decomposed into various products, such as gluconic acid, which was then oxidized to 2-keto-D-gluconic acid (2-KGA) and 5-keto-D-gluconic acid (5-KGA) (32). During fermentation, the tartaric acid content gradually decreased, and the reduction rate was about 37.42%. In conclusion, lactic acid bacteria fermented ginkgo biloba kernel juice could transform and generate a variety of organic acids. Organic acid was the base of taste substance of ginkgo biloba kernel juice, and was the precursor of many flavor substances. Volatile flavor substances such as acids, alcohols, and aldehydes will be produced in the process of microbial metabolism. Therefore, we evaluated the changes of flavor substances before and after fermentation (Table 5).

**TABLE 3:** Changes in the content of different organic acids during fermentation (μg/mL).

Fermentation time (h)	0	4	12	24	48
Oxalic acid	43.01 ± 0.67^a^	37.64 ± 0.12^b^	44.64 ± 1.69^a^	43.65 ± 0.68^a^	37.63 ± 1.59^b^
Tartaric acid	632.34 ± 19.98^a^	536.93 ± 4.41^b^	545.08 ± 32.27^b^	514.06 ± 9.97^b^	395.72 ± 2.61^c^
Quinic acid	584.52 ± 0.68^a^	448.64 ± 22.36^c^	440.38 ± 13.16^c^	229.27 ± 15.61^d^	497.96 ± 1.6^b^
Pyruvate	80.27 ± 0.27^b^	83.12 ± 2.07^a^	84.51 ± 1.45^a^	61.39 ± 1.02^c^	56.68 ± 1.56^d^
Malic acid	926.91 ± 5.35^b^	711.77 ± 10.41^d^	866.05 ± 14.87^c^	1019.7 ± 14.75^a^	692.95 ± 3.51^d^
Shikimic acid	13 ± 0.7^e^	26.73 ± 1.36^d^	73.78 ± 1.45^c^	90.49 ± 0.48^b^	114.15 ± 7.22^a^
Lactic acid	403.33 ± 17.77^c^	530.72 ± 25.16^b^	731.24 ± 18.87^a^	695.37 ± 3.88^a^	722.58 ± 29.76^a^
Citric acid	43.77 ± 0.55^a^	37.28 ± 1.96^b^	12.32 ± 0.25^d^	35.37 ± 3.95^b^	32.19 ± 0.83^c^
Fumaric acid	0.04 ± 0.01^c^	0.01 ± 0.01^d^	0.01 ± 0.01^d^	0.26 ± 0.01^a^	0.23 ± 0.01^b^
Succinic acid	163.04 ± 1.67^b^	39.21 ± 0.96^c^	57.12 ± 1.74^c^	551.28 ± 31.12^a^	565.83 ± 19.21^a^

Results are expressed as the mean ± standard deviation of three replicates. The mass concentrations of substances in different time periods are represented by different letters, indicating significant differences (*p* < 0.05).

### Study on the change of total protein content and free amino acid profile of Y2 fermentation

Lactic acid bacteria could degrade macromolecular proteins into small peptide chains, amino acid proteases and peptidases through their own reproductive metabolism and hydrolysis during fermentation (33). Under the catalytic reaction, the proteases could degrade the protein into smaller peptides that are degraded into the small-molecule free amino acids by the action of the peptidase (34). The total protein content of ginkgo biloba kernel juice decreased continuously during the fermentation process, and approximately 67.51% of the metabolism was consumed at the end of fermentation (Table 2).

Among all free amino acids, glutamic acid had the highest content (74.22 ± 0.01 μg/mL), followed by arginine (55.68 ± 0.01 μg/mL) and alanine (20.83 ± 0.05 μg/mL) (Table 4). Glutamate, free histidine and alanine levels decreased significantly after fermentation. During the fermentation process, under the action of lactic acid bacteria decarboxylase, Glu is converted into γ-aminobutyric acid, and Asp is converted into Ala (35). The combination of aspartic acid, glutamic acid and Na^+^ makes the sample umami, which would give ginkgo biloba juice rich flavor substances. In addition, the content of aromatic amino acids (Phe) decreased significantly after fermentation, while the content of another aromatic tyrosine (Tyr) increased, owing to the effect of amino acid invertase on the fermentation process (36). The content of branched-chain amino acids (Leu, Ile) also decreased significantly after fermentation, then were metabolized into keto acids, alcohols and fatty acids through amino acid converting enzymes. Phe and Thr could be converted into benzyl alcohol, phenylethanol and acetaldehyde with certain flavor under the action of an amino acid converting enzyme. Arginine is converted to citrulline by the enzyme arginine deiminase during lactic acid fermentation, which is further broken down into ornithine and free ammonia (37).

**TABLE 4:** Changes in the content of different free amino acids in fermented and unfermented (μg/mL).

Fermentation time (h)	0	48
Asp	4.61 ± 0.06^a^	2.71 ± 0.01^b^
Thr	5.37 ± 0.04^a^	3.39 ± 0.01^b^
Ser	6.10 ± 0.01^a^	2.32 ± 0.01^b^
Glu	74.22 ± 0.01^a^	67.51 ± 0.02^b^
Gly	1.72 ± 0.02^b^	7.62 ± 0.02^a^
Ala	20.83 ± 0.05^a^	7.58 ± 0.03^b^
Cys	1.09 ± 0.01^c^	2.34 ± 0.04^a^
Val	3.81 ± 0.05^b^	5.88 ± 0.02^a^
Met	0.43 ± 0.01^b^	1.58 ± 0.03^a^
Ile	3.68 ± 0.03^a^	1.82 ± 0.02^b^
Leu	6.40 ± 0.01^a^	3.43 ± 0.06^b^
Tyr	10.72 ± 0.04^b^	11.79 ± 0.03^a^
Phe	3.63 ± 0.06^a^	3.42 ± 0.03^b^
Lys	5.18 ± 0.03^b^	6.92 ± 0.06^a^
NH_3_	0.48 ± 0.01^b^	0.58 ± 0.03^a^
His	42.45 ± 0.01^a^	28.40 ± 0.01^b^
Arg	55.68 ± 0.01^b^	72.28 ± 0.02^a^

Results are expressed as the mean ± standard deviation of three replicates. The mass concentrations of substances in different time periods are represented by different letters, indicating significant differences (*p* < 0.05).

**TABLE 5:** Changes in the content of different phenolic acids during fermentation (μg/mL).

Fermentation time (h)	0	4	12	24	48
Catechin	7.32 ± 0.06^c^	9.55 ± 0.18^a^	7.52 ± 0.12^b^	6.70 ± 0.01^d^	6.73 ± 0.06^d^
Hippuric acid	5.48 ± 0.06^d^	16.47 ± 0.03^b^	4.71 ± 0.04^e^	15.52 ± 0.34^c^	17.8 ± 0.71^a^
Homovanillic acid	2.60 ± 0.03^b^	3.36 ± 0.01^a^	2.31 ± 0.06^d^	2.51 ± 0.01^c^	2.53 ± 0.04^c^
p-coumaric acid	0.16 ± 0.01^c^	0.10 ± 0.01^e^	0.14 ± 0.01^d^	0.18 ± 0.01^b^	0.25 ± 0.01^a^
Phloretin	0.48 ± 0.02^a^	0.37 ± 0.01^b^	0.34 ± 0.01^b^	0.35 ± 0.01^b^	0.33 ± 0.01^c^
p-vinyl guaiacol	1.58 ± 0.01^a^	0.05 ± 0.46^d^	0.40 ± 0.01^c^	1.16 ± 0.01^b^	0.59 ± 0.01^c^
Proanthocyanidin B2	125.92 ± 0.56^b^	152.28 ± 0.92^a^	111.86 ± 1.5^c^	98.79 ± 0.17^d^	97.76 ± 0.40^d^
Chlorogenic acid	2.38 ± 0.01^d^	3.64 ± 0.08^a^	2.43 ± 0.07^d^	3.35 ± 0.05^b^	2.86 ± 0.24^c^
p-hydroxybenzoic acid	9.64 ± 0.09^b^	12.35 ± 0.18^a^	8.04 ± 0.02^c^	7.65 ± 0.09^d^	7.91 ± 0.04^c^
Syringic acid	0.50 ± 0.01^a^	0.71 ± 0.02^a^	0.40 ± 0.01^b^	0.48 ± 0.01^a^	0.50 ± 0.01^b^
Phloridic acid (para-hydroxypropionic acid)	0.73 ± 0.03^b^	0.77 ± 0.02^b^	0.58 ± 0.01^d^	0.68 ± 0.01^c^	1.09 ± 0.05^a^
Ferulic acid	0.22 ± 0.01^e^	0.32 ± 0.01^d^	0.37 ± 0.01^c^	0.46 ± 0.01^b^	0.61 ± 0.04^a^
Cinnamic aldehyde	0.11 ± 0.01^a^	0.12 ± 0.01^a^	0.11 ± 0.01^a^	0.10 ± 0.01^b^	0.10 ± 0.01^c^
Gallic acid	1.16 ± 0.01^c^	1.51 ± 0.01^b^	1.11 ± 0.01^c^	1.10 ± 0.03^c^	1.87 ± 0.10^a^
Protocatechuic acid	1.88 ± 0.02^c^	2.75 ± 0.04^b^	1.81 ± 0.01^d^	1.67 ± 0.04^e^	3.62 ± 0.05^a^
Caffeic acid	0.45 ± 0.02^d^	0.58 ± 0.05^b^	0.52 ± 0.03^c^	0.61 ± 0.02^b^	0.73 ± 0.06^a^
Rutin	0.11 ± 0.11^b^	0.09 ± 0.73^c^	0.24 ± 0.01^a^	0.11 ± 0.01^b^	0.21 ± 0.01^a^
Quercitrin (quercetin-3-rhamnoside)	0.01 ± 0.01^d^	0.03 ± 0.01^a^	0.01 ± 0.01^c^	0.01 ± 0.01^e^	0.02 ± 0.01^b^

Results are expressed as the mean 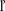 standard deviation of three replicates. The mass concentrations of substances in different time periods are represented by different letters, indicating significant differences (*p* < 0.05).

The total free amino acid content increased by approximately 6.88% after 48 h of fermentation. The effects of lactic acid fermentation on free amino acid profiles were studied using principal component analysis (PCA). In principal component analysis, PC1 and PC2 accounted for 98.2 and 1.8% of the data variance, respectively. As shown in the composition diagram (Figure 3C), the samples of unfermented ginkgo biloba kernel juice were located on the positive side of PC1, and the samples fermented for 48 h were distributed on the negative side. This indicated that lactic acid bacteria had a significant effect on the change in free amino acid content during the fermentation of ginkgo biloba kernel juice. Specific changes in the free amino acids were observed through in the loading diagram (Figure 3D). Phe, Thr, Leu, Ile, Asp, Ser, Ala, and free histidines were all positively correlated with PC1, indicating that these amino acids would be affected by the fermentation process. Whereas, Lys, Val, Met, Gly, Cys, Tyr, and Arg were all negatively correlated with PC2, indicating that their content increased with the fermentation time.

### Effect of Y2 fermentation on the structure of phenolic substances

Ginkgo kernels were also rich in a variety of phenolic substances, which led to the production of many secondary metabolites during fermentation, including phenolic acids, stilbenes, lignins, and aromatic compounds (38). Therefore, it is necessary to study the effect of LAB fermentation on the phenolic components in ginkgo biloba kernel juice.

As shown in Table 1, the total phenolic content of ginkgo biloba kernel juice at the beginning of fermentation was 116.51 ± 0.51 mg/L. It decreased significantly within 4 h of fermentation, and then increased slowly. After 48 h of fermentation, the total phenolic content of the samples was approximately 9.72% higher than that of the unfermented samples. Procyanidin B2 is an oligomer, dimer and polymer of catechin (39). The highest content of flavonoids in ginkgo kernel was about 7.32 ± 0.06 μg/mL of catechin and about 125.92 ± 0.56 μg/mL of procyanidin B2 (Table 5). Procyanidin B2 increased by approximately 20.93%, and catechin increased by approximately 30.46% after 4 h of fermentation. The increase in the total flavonoid content could result in an increase in the antioxidant activity of the fermented samples. In the process of fermenting ginkgo biloba kernel juice, the improvement in antioxidant activity was highly correlated with the transformation of phenolic substances (40). Previous studies have reported that phenolic compounds could be used as reducing agents as well as free radical scavengers and singlet oxygen quenchers (41). Phenols could be converted into each other, and the antioxidant activity of the fermentation broth can be improved (42). It was also shown that the antioxidant capacity of phenolic substances in ginkgo biloba kernel juice was significantly improved after Y2 fermentation (Figures 2C,D). There is also a certain relationship between FRAP and phenolic content, and the presence of phenolic substances can effectively reduce Fe^3+^ to Fe^2+^ form (43). Therefore, the increase in phenolic substances was an important factor affecting the antioxidant activity of fermentation.

As shown in Figures 3E,F, principal component analysis (PCA) was used to analyze the effect of Y2 fermentation on phenolic components in ginkgo biloba kernel juice. After 48 h of fermentation, phloridzin, epicatechin and gallate in the samples were metabolized to form gallic acid and phloretin with a strong antioxidant quality (44). *L. plantarum* fermentation could produce some phenolic acid decarboxylases, such as ferulic acid. These extracellular enzymes act on phenolic components, and from a chemical point of view, glycosylation, methylation and other types of substitutions are carried out, resulting in structural changes. This lead to the mutual conversion of phenolic substances in the sample, thereby changing their oxidative activity (45). The p-hydroxybenzoic acid content decreased by approximately 17.95% after fermentation. Further, the content of chlorogenic acid was the highest at 4 h of fermentation (3.64 ± 0.08 μg/mL), and it increased by about 20.17% at the end of fermentation. The increase in chlorogenic acid content leads to an increase in pharmacological functions, such as antioxidant, antiviral, anti-cancer and antibacterial capacities (46). During the fermentation period of 4–8 h, the content of chlorogenic acid gradually decreased, potentially under the action of extracellular enzyme hydrolysis, which indirectly led to an increase in caffeic acid, p-coumaric acid and ferulic acid. Phenolic acids not only have various pharmacological effects such as anti-inflammatory, antioxidant, anti-mutation, and anti-cardiovascular disease, but also have co-color effects (such as ferulic acid), which can enhance the stability of their own colors after fermentation (47). There are few studies on the volatile flavor compounds after fermentation, and further research is needed.

### Study on volatile flavor compounds by fermentation of Y2

**FIGURE 4:**
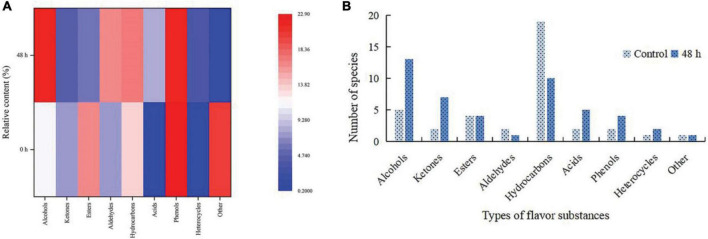
Changes in relative content **(A)** and number of species **(B)** of volatile flavor compounds in Ginkgo biloba kernel juice fermented by Y2 for 48 h.

A total of 64 volatile flavor compounds were identified in ginkgo biloba kernel juice before and after fermentation, including three kinds of aldehydes, 18 kinds of alcohols, 9 kinds of ketones, 8 kinds of esters, 29 kinds of hydrocarbons, 6 kinds of phenols, 3 kinds of heterocycles, 7 kinds of acids, and two other types. From the analysis results, the fermented samples and the unfermented samples were quite different, and the similarities of volatile flavor substances were low. The relative content and species of ginkgo biloba kernel juice in alcohols, acids, and heterocyclic compounds were significantly increased after 48 h of fermentation (Figure 4).

The main aroma substances in unfermented ginkgo biloba kernel juice were 2-ethyl-1-hexanol, 1-octanol, and n-octanol (Table 6). The total relative content of volatile flavor compounds increased by 125.48% after fermention. Among them, the total relative content of alcohol increased by about 104.69%, potentially due to the emergence of new generation of various volatile flavor substances. After 48 h of fermentation, the contents of 1-octanol and n-octanol increased significantly, the contents of 2-ethyl-1-hexanol and 1-octanol decreased, and 1-heptanol, 1-octen-3-ol and 2-octen-1-ol newly formed. The relatively high contents of newly generated alcohols were geraniol (1.81%), 1-octen-3-ol (2.65%) and 1-heptanol (3.24%). These alcohols were mainly produced by metabolism during microbial processes, degradation of unsaturated fatty acids, and reduction of carbon-based compounds during fermentation. Aldehydes are unstable compounds, and the relative content of aldehydes increased by about 126.5%, among which the highest content was 16.15% 2,5-dimethylbenzaldehyde. The relative content of hydrocarbons increased by about 32.92%, while the types of hydrocarbons decreased after fermentation. Meanwhile, the relative content of newly generated aromatic compounds was higher, of which 1,6-dimethyl-4-(1-methylethyl)-naphthalene accounted for about 2.18% of the total relative content after fermentation. The increase of total content of alcohols and esters in ginkgo biloba kernel juice could mpart stronger fruity and floral aromas (48). Under the metabolic activity of lactic acid bacteria, more aldehydes are reduced to alcohols and acids, and the increase of alcohols will increase the corresponding esters, which is also the reduction of aldehydes in fermented ginkgo biloba kernel juice, while alcohol, the reasons for the increase in esters (49). The content of acids increased by about 786.01%, and the relative content of acids increased significantly. The strain Y2 produced a large amount of acids during the fermentation process, among which the new acids were 2.16% methoxy-acetic acid, 1.02% dodecanoic acid, 0.29% 2-methylbutyric acid, 0.24% 2-ethylhexanoic acid. Among them, the relative contents of L-lactic acid and propionic anhydride decreased after the fermentation, while the relative content of acetic anhydride was high (4.18%). Meanwhile, the relative content of phenols changed scarcely, and eugenol (7.48%) and 3-ethylphenol (0.29%) were newly formed at the end of fermentation. The former was produced by the reaction of guaiacol and allyl alcohol, and had strong antibacterial activity. The latter had an aromatic odor and was widely used in organic synthesis and as a solvent (50). Future research will focus on the transformation mechanism between these molecules and their effect on the flavor of ginkgo biloba kernel juice.

**TABLE 6:** Relative content change of fermented and unfermented flavor substances (%).

Component name	CAS no.	Retention time (min.)	Relative content (%)	
			0 h	48 h
**Alcohols**				
Ethanol	64-17-5	1.456	2.46	3.01
2-ethyl-1-Hexanol,	104-76-7	11.792	7.18	3.1
1-Octanol	111-87-5	13.224	0.68	1.58
1-Dodecanol	112-53-8	24.529	0.13	–
3-methyl-3-Buten-1-ol,	763-32-6	2.885	–	0.85
1-Pentanol	71-41-0	2.905	–	0.46
1-Hexanol	111-27-3	6.292	–	2.39
1-Heptanol	111-70-6	9.807	–	3.24
1-Octen-3-ol	3391-86-4	10.139	–	2.65
2-Octen-1-ol	22104-78-5	13.147	–	0.36
1-Nonanol	143-08-8	16.353	–	1.40
Geraniol	106-24-1	18.764	–	1.80
2,4-Decadien-1-ol	14507-02-9	20.672	–	0.27
1,3,4,5,6,8a-hexahydro-4,7-dimethyl-1-(1-methylethyl)-, (1S,4R,4aS,8aR)-4a(2H)-Naphthalenol,	19912-67-5	28.218	–	0.28
**Ketones**				
Acetone	67-64-1	1.52	7.16	1.78
7-methyl-2-Oxepanone,	2549-59-9	10.924	0.25	–
trans-.beta.-Ionone	79-77-6	18.875	–	0.62
3,4,4a,5,6,7-hexahydro-1,1,4a-trimethyl-2(1H)-Naphthalenone,	4668-61-5	19.475	–	1.13
dihydro-5-pentyl-2(3H)-Furanone,	104-61-0	21.699	–	0.40
2-Tridecanone	593-08-8	25.056	–	0.41
2-Pentadecanone	2345-28-0	29.68	–	0.11
7,9-Di-tert-butyl-1-oxaspiro(4,5)deca-6,9-diene-2,8-dione	82304-66-3	34.224	–	0.08
**Esters**				
Ethyl Acetate	141-78-6	1.889	4.54	–
Methyl anthranilate	134-20-3	21.155	8.63	3.97
Acetic acid, decyl ester	112-17-4	22.936	2.80	1.94
2-Undecanol, acetate	14936-67-5	23.513	0.22	0.13
Decanoic acid, 3- hydroxy-, methyl ester	56618-58-7	24.241	–	0.09
**Aldehydes**				
Hexanal	66-25-1	4.082	3.88	–
Nonanal	124-19-6	14.25	3.25	–
2,5-dimethyl-Benzaldehyde	5779-94-2	17.594	–	16.15
**Hydrocarbons**				
n-Hexane	110-54-3	1.812	0.61	–
Trichloromethane	67-66-3	1.906	0.47	0.17
3-methyl-Butanal	590-86-3	2.12	0.47	–
2,3,4-trimethyl-Hexane	921-47-1	6.014	0.82	–
Undecane	1120-21-4	12.748	2.45	–
2,4-dimethyl-Decane	2801-84-5	12.926	1.22	–
Dodecane	112-40-3	17.198	0.83	0.58
Longifolene	475-20-7	22.886	0.40	0.15
(1S,4S,4aS)-1-Isopropyl-4,7-dimethyl-1,2,3,4,4a,5-hexahydronaphthalene	267665-20-3	24.009	0.29	–
1-Isopropyl-4,7-dimethyl-1,2,3,4,5,6-hexahydronaphthalene	16729-00-3	24.579	0.33	–
cis-Muurola-4(15),5-diene	157477-72-0	25.046	0.41	–
Pentadecane	629-62-9	25.139	0.25	–
1-Isopropyl-4,7-dimethyl-1,2,3,5,6,8a-hexahydronaphthalene	16729-01-4	25.777	1.72	–
Trans-Calamenene	73209-42-4	25.783	1.95	–
Cubenene	29837-12-5	25.998	0.35	–
Alpha-calacorene	21391-99-1	26.256	0.52	8.79
4-Isopropyl-6-methyl-1-methylene-1,2,3,4-tetrahydronaphthalene	50277-34-4	26.736	0.09	–
Alpha-corocalene	20129-39-9	28.091	0.05	2.2
1,3,4,5,6,8a-hexahydro-4,7-dimethyl-1-(1-methylethyl)-, (1S,4R,4aS,8aR)-4a(2H)-Naphthalenol	19912-67-5	28.221	0.75	–
1,5-dimethy l-Cyclopentene	16491-15-9	18.59	–	0.55
Tridecane	629-50-5	20.018	–	1.62
*Cis*-Calamenene	72937-55-4	25.78	–	0.73
2-Acetoxytridecane		28.158	–	0.15
1,6-dimethyl-4-(1-methylethyl)-Naphthalene	483-78-3	29.254	–	2.18
**Acids**				
L-Lactic acid	79-33-4	3.797	0.20	–
Propanoic acid, anhydride	123-62-6	10.142	0.69	–
Methoxy-acetic acid	625-45-6	2.271	–	2.16
Acetic anhydride	108-24-7	2.285	–	4.18
2-methyl-Butanoic acid	116-53-0	5.866	–	0.29
2-ethyl-Hexanoic acid	149-57-5	14.837	–	0.24
Dodecanoic acid	143-07-7	26.628	–	1.02
**Phenols**				
Alpha-cubebene	17699-14-8	21.397	2.27	–
2,4-Di-tert-butylphenol	96-76-4	25.445	20.53	13.47
3-ethyl-Phenol	620-17-7	16.273	–	0.29
Eugenol	97-53-0	21.568	–	7.48
*Trans*-Isoeugenol	5932-68-3	23.966	–	0.37
**Heterocycles**				
Decane-rel-(1R,2S,6S,7S,8S)-8-Isopropyl-1-methyl-3-methylenetricyclo[4.4.0.02,7]	18252-44-3	22.47	0.27	–
2,3-dihydro-1,1,5,6-tetramethyl-1H-Indene,	942-43-8	16.313	–	0.11
3,5,6,8a-tetrahydro-2,5,5,8a- tetramethyl-,trans-2H-1-Benzopyran,	41678-29-9	20.434	–	3.41
**Other**				
Oxime-, methoxy-phenyl		7.614	20.49	1.65

”–” is not detected.

### Effect of Y2 fermentation on the content of 4′-*O*-methylpyridoxine

**FIGURE 5:**
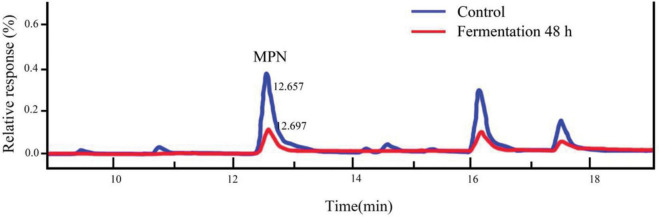
Changes between fermented and unfermented MPN content.

4′-O-methylpyridoxine (MPN), also known as ginkgo toxin, is an anti-vitamin B6 compound, and one of the toxic components in ginkgo kernels. If ingested in large amounts, adverse symptoms, such as vomiting, clonic seizures, and loss of consciousness could occur (51). Relevant studies had shown that MPN could be effectively converted into 4′-*O*-methylpyridoxine-5-glycoside (MPNG) after heating, microwave irradiation and boiling (52). In this study, the MPN content in ginkgo biloba kernel juice was identified, by comparison with standard chemicals determined by high performance liquid phase (HPLC) analysis (Figure 5). The content of MPN was reduced by roughly 82.25% after fermentation for 48 h, indicating that strain Y2 could effectively gradate MPN. Relevant studies have shown that lactic acid bacteria have the function of adsorbing toxins during the fermentation process. During the growth of lactic acid bacteria, a large number of polysaccharides and peptidoglycan were produced on the cell wall, which played a key role in adsorbing the toxic substance 4′-O-methylpyridoxine in ginkgo biloba kernel juice (53). It also had been reported that MPN could be phosphorylated to form 4′-*O*-methylpyridoxine-5′-phosphate (MPNP) under the action of phosphoric acid produced during LAB fermentation (54). However, there are few studies on the degradation mechanism of 4′-*O*-methylpyridoxine content by LAB fermentation, and we will further investigate how strain Y2 bio-transformed MPN.

## Conclusion

This work showed that LAB fermentation was a potential way to improve the beneficial value and safety of ginkgo biloba kernel juice. The total relative content of volatile flavor substances increased by about 125.48%, and 24 new substances were added. The biotransformation and biosynthesis content of the phenols was increased, and the total phenolic concentration increased by about 9.72%, which indirectly led to the enhance of antioxidant capacity of the fermentation. The increase of the aromatic amino acids and volatile flavor substances reflected the metabolism of lactic acid bacteria in fruit juice. In addition, 4′-*O*-methylpyridoxine, as one of the toxic substances in ginkgo biloba kernel, showed a degradation rate above 82.25%, and the total ginkgo acid content in the final product was less than 41.53 mg/L. As few reports about the degradation of 4′-*O*-methylpyridoxine by fermentation, further studied will focus on the degradation mechanism. Our results showed that the LAB fermented ginkgo biloba kernel juice had broad application prospects.

## Data availability statement

The original contributions presented in this study are included in the article/supplementary material, further inquiries can be directed to the corresponding author/s.

## Author contributions

JY and YS performed the experiments and analyzed the data. JC, YC, HZ, and TG drafted the manuscript. FX and SP analyzed and discussed the data. YT and JL contributed to the writing—review, editing, and funding acquisition. All authors have read and agreed to the published version of the manuscript.
